# Research on the Pathogenesis of Cognitive and Neurofunctional Impairments in Patients with Noonan Syndrome: The Role of Rat Sarcoma–Mitogen Activated Protein Kinase Signaling Pathway Gene Disturbances

**DOI:** 10.3390/genes14122173

**Published:** 2023-12-03

**Authors:** Natalia Braun-Walicka, Agnieszka Pluta, Tomasz Wolak, Edyta Maj, Agnieszka Maryniak, Monika Gos, Anna Abramowicz, Aleksandra Landowska, Ewa Obersztyn, Jerzy Bal

**Affiliations:** 1 The Department of Medical Genetics, Institute of Mother and Child, 01-211 Warsaw, Poland; 2 The Bioimaging Research Center, World Hearing Center, Institute of Physiology and Pathology of Hearing, Kajetany, 05-830 Nadarzyn, Poland; 3 The Faculty of Psychology, University of Warsaw, 00-183 Warsaw, Poland; 4 2nd Department of Clinical Radiology, Medical University of Warsaw, 02-097 Warsaw, Poland

**Keywords:** Noonan syndrome, cognitive impairments, functional magnetic resonance imaging, intelligence tests

## Abstract

Noonan syndrome (NS) is one of the most common genetic conditions inherited mostly in an autosomal dominant manner with vast heterogeneity in clinical and genetic features. Patients with NS might have speech disturbances, memory and attention deficits, limitations in daily functioning, and decreased overall intelligence. Here, 34 patients with Noonan syndrome and 23 healthy controls were enrolled in a study involving gray and white matter volume evaluation using voxel-based morphometry (VBM), white matter connectivity measurements using diffusion tensor imaging (DTI), and resting-state functional magnetic resonance imaging (rs-fMRI). Fractional anisotropy (FA) and mean diffusivity (MD) probability distributions were calculated. Cognitive abilities were assessed using the Stanford Binet Intelligence Scales. Reductions in white matter connectivity were detected using DTI in NS patients. The rs-fMRI revealed hyper-connectivity in NS patients between the sensorimotor network and language network and between the sensorimotor network and salience network in comparison to healthy controls. NS patients exhibited decreased verbal and nonverbal IQ compared to healthy controls. The assessment of the microstructural alterations of white matter as well as the resting-state functional connectivity (rsFC) analysis in patients with NS may shed light on the mechanisms responsible for cognitive and neurofunctional impairments.

## 1. Introduction

Noonan syndrome (NS) is the most common RASopathy and is a genetic condition inherited in an autosomal dominant manner, although an autosomal recessive inheritance associated with biallelic pathogenic *LZTR1* mutations has been confirmed [1]. 

Among the typical clinical symptoms of Noonan syndrome are distinctive facial features, short stature, congenital heart defects (particularly pulmonary valvar stenosis), chest malformations, pigmented skin lesions, osteoarticular defects, visual impairments, variable developmental delay, and cognitive impairments. An increased risk of malignancy including hematoproliferative diseases has been reported [2,3,4].

Germline pathogenic variants in genes encoding proteins in the RAS/MAPK signaling pathway (Rat Sarcoma–Mitogen Activated Protein Kinase) play an essential role in the pathogenesis of Noonan syndrome [5]. The RAS/MAPK signaling pathway is one of the basic cellular signaling cascades responsible for signal transduction from the outer cell membrane to the nucleus, playing an essential role in the regulation of cell function including cellular proliferation, differentiation, and survival. Apart from human developmental syndromes, RAS/MAPK pathway impairments are one of the most common causative factors in oncogenesis [5,6,7].

In recent years, there has been an increasing interest in cognitive and social functioning impairments in NS patients. Recent studies have revealed that weaknesses in cognitive functioning are much more frequent in patients with Noonan syndrome than in the general population [8,9,10,11,12].

Van der Burgt et al. conducted one of the first studies on cognitive functioning in NS patients. Standardized tests of intellectual disability revealed remarkably lower mean full-scale IQs in children diagnosed with NS (*n* = 35, age range 7–18 years) [8]. According to the study of Alfieri et al. on neurobehavioral impairments in individuals with RASopathy (*n* = 70, age range 2.3–28 years), including Noonan syndrome (*n* = 38), a comprehensive psychopathological assessment is required in these patients [9]. In the study of Pierpont et al., children with NS demonstrated higher risks for ADHD and functional impairments in comparison to their unaffected siblings [10]. 

Based on the current knowledge regarding the involvement of the RAS/MAPK pathway in brain development, including synaptic plasticity and neuronal functions, it is highly probable that cognitive deficits may be associated with mutations in particular genes, leading to the dysregulation of the RAS/MAPK pathway [11,12]. 

Cognitive deficits may result from structural and/or functional abnormalities in particular brain regions or networks caused by disturbances in the RAS/MAPK signaling cascade. It is, therefore, possible that patients with Noonan syndrome may demonstrate structural abnormalities detected by VBM (voxel-based morphometry), a neuroimaging technique involving a voxel-wise comparison of regional gray matter volume [13,14]. Reductions in white matter connectivity are another eventuality in patients with Noonan syndrome that may be revealed using DTI (diffusion tensor imaging). The most common DTI measurement is fractional anisotropy (FA), which reflects the degree of diffusion anisotropy and is sensitive to microstructural changes [15]. Finally, changes in the resting-state functional connectivity of the brain revealed using rs-fMRI (resting-state functional magnetic resonance imaging) may also be expected [16,17]. There is increasing evidence that synchronized spontaneous brain activity is essential for normal brain functions. Resting-state alterations including decreased activity and connectivity within various functional networks have been observed in numerous neurodevelopmental and neurodegenerative diseases [18]. 

The aim of our research project was to verify the hypothesized potential role of disturbances in the RAS/MAPK signaling cascade in the pathomechanism of cognitive impairments, as well as functional and structural brain abnormalities examined using region-based morphometry, DTI, and rs-fMRI in patients with molecularly confirmed Noonan syndrome. 

## 2. Materials and Methods

Participants were recruited from a cohort of families who were followed up at the Department of Medical Genetics at the Institute of Mother and Child (Warsaw, Poland). All participants were evaluated by an experienced clinical geneticist. Clinical diagnosis of Noonan syndrome was based on diagnostic criteria developed by van der Burgt in 2007 and confirmed with molecular testing [19]. 

Patients with a molecularly confirmed pathogenic mutation in one of the RAS/MAPK pathway genes responsible for the clinical expression of Noonan syndrome were excluded from the project in the case of concurrent:

1. Severe or profound intellectual disability.
2. History of medical disease known to affect brain structure (e.g., epilepsy, moderate/severe head injury, head trauma).
3. Hearing loss.

The study group consisted of 37 patients with molecularly confirmed Noonan syndrome between the ages of 5.0 and 48.0 years (mean age 15.73), including 14 (37.8%) female patients and 23 (62.2%) male patients. The patient cohort included 18 individuals with *PTPN11* mutations, 9 with *SOS1* mutations, 5 with *RIT1* mutations, 2 with *RAF1* mutations, 1 patient with *CBL* mutation, 1 with *KRAS* mutation, and 1 with *SHOC2* mutation. The mean age of patients with *PTPN11* mutations was 15.23 years (range: 5.0–47.0), and that of patients with *SOS1* mutation was 15.07 years (range: 5.0–42.0).

The control group consisted of 27 age- and sex-specific normally developing siblings of recruited patients with Noonan syndrome, between the ages of 3.0 and 38.0 years (mean age 13.60), including 14 (51.9%) female patients and 13 (48.1%) male patients. This control group was meant to exclude the impact of family socioeconomic status. which is extremely important in the case of investigations of cognitive impairments in patients with genetic disorders.

### 2.1. Cognitive Assessment

Participants were investigated with a detailed psychological evaluation, administered by a trained psychologist (AP) using standardized procedures. 

The examination of cognitive abilities was conducted with the Stanford Binet Intelligence Scales, Fifth Edition (SB5) [20]. The SB5 is a standardized test of intellectual abilities for children and adults between the ages of 2 and 85 years. It provides a general ability score (full scale IQ, FSIQ), and five indices measuring fluid reasoning (FR), knowledge (KN), quantitative reasoning (QR), visual spatial processing (VS), and working memory (WM), divided into two domains, verbal (VIQ) and non-verbal (NVIQ). The test shows high reliability. Alpha coefficients for the full scale ranged from 0.95 to 0.98, and they were similar in the verbal and nonverbal area (0.91–0.95) and for individual factors (0.88–0.91). The test shows high stability over time [20]. 

Outliers were examined using box plot methods, implemented in the R software (v4.1.0) [21]. There were no extreme outliers. The Shapiro–Wilk test was used to assess the normality of each variable’s distribution, and Levene’s test was used to assess the homogeneity of variance in the different groups. One-way analysis of variance (ANOVA) and Student’s t-test were used to compare the NS and HC groups. 

Secondly, we compared cognitive functioning of subjects with *PTPN11* and *SOS1* mutations. As the groups differed in size (18 vs. 9 subjects), our first step was to select two equinumerous groups with participants balanced on age. We chose to conduct matching using the MatchIt package in R with the nearest neighbor algorithms [22]. The final sample resulted in two successfully balanced groups (*n* = 9 each) according to age (*PTPN11*: mean age 15.1, SD = 11.1, age range: 5.6–42.4; *SOS1*: mean age 15, SD = 11.1, age range: 5.8–41.9). We thereafter applied a nonparametric Wilcoxon signed-rank test to compare cognitive measures between the two.

### 2.2. Resting-State Functional Connectivity

#### 2.2.1. Rs-fMRI Data Acquisition

Fifty one (28—NS, 23—HC) subjects took part in the rs-fMRI examination.

Rs-fMRI data were acquired at the Bioimaging Research Center of IPPH in Kajetany/Warsaw, Poland. Rs-fMRI examination was conducted using a 3T Siemens Prisma Fit scanner equipped with a 20-channel head coil. 

The EPI sequence parameters were as follows: time of repetition (TR) = 1500 [ms]; time of echo (TE) = 28 [ms]; flip angle (FA) = 90°; voxel size = 2 × 2 × 2.4 mm; imaging matrix = 96 × 96; no. of slices = 48; time of acquisition (TA) = 10:08 min; 400 data points. 

Participants were instructed during scanning to relax with their eyes open and not to think of anything in particular. 

The structural T1 MR sequence had the following parameters: TR = 2300 [ms]; TE = 2.26 [ms]; time of inversion (TI) = 900 [ms]; flip angle (FA) = 8°; field of view (FOV) = 20.8 × 23.0 [cm]; matrix = 232 × 256; voxel size = 0.9 × 0.9 × 0.9 [mm]; pixel bandwidth = 310 Hz/pix; no. of slices = 192; TA = 5:11 min.

#### 2.2.2. Data Preprocessing

A standard preprocessing pipeline was applied in CONN 21a (Connectivity Toolbox), which uses functions from the Statistical Parametric Mapping 12 (SPM12 (7771)) software. Preprocessing of the functional data included slice timing correction, motion correction, scrubbing, linear detrending, band-pass filtering (0.01 Hz < f < 0.1 Hz), co-registration to individual T1 structural scans, spatial normalization to standard space (MNI template), and spatial smoothing (6 mm Gaussian kernel). Each subject’s structural scan was segmented into gray matter, white matter, and cerebrospinal fluid (CSF) tissue classes using the unified segmentation approach implemented in SPM12. In addition, the Artifact Detection Tool (available online: https://www.nitrc.org/projects/artifact_detect/ (accessed on 20 October 2015)) was used to measure motion artifacts in all individuals. Linear regression of confounding effects was applied including: the 5 most significant signal components from the white matter and CSF, motion parameters obtained in the realignment step, volumes that showed movement exceeding 0.5 mm from the scrubbing step (in the ART toolbox with conservative settings, 95th percentile in a normative sample) and 10 first scans (effect of rest). 

#### 2.2.3. First-Level and Second-Level Analyses

Functional connectivity (FC) measures were computed between 32 regions of interest (ROIs) from classical networks—Default Mode Network (4 ROIs), Sensori-Motor (2 ROIs), Visual (4 ROIs), Salience/Cingulo-Opercular (7 ROIs), Dorsal Attention (4 ROIs), Fronto-Parietal/Central Executive (4 ROIs), Language (4 ROIs), and Cerebellar (2 ROIs)—by computing bivariate Pearson’s correlation measures between the extracted mean BOLD signal time courses of each pair of ROIs. 

Next, we compared FC between groups using two-tailed paired *t*-tests. In order to account for age differences between subjects, we included age as a covariate in the model. 

In order to correct for multiple comparisons, we applied the Spatial Pairwise Clustering (SPC) approach [23]. In our analyses, the following SPC parameters were used: cluster threshold: *p* < 0.05, cluster-level p-FDR corrected (SPC mass/intensity); connection threshold: *p* < 0.01, *p* uncorrected.

### 2.3. Diffusion Tensor Imaging

Fifty seven (34—NS, 23—HC) subjects took part in the DTI examination. 

#### 2.3.1. Data Acquisition

Diffusion-weighted images were collected using a 3T Prisma Scanner and the gradient echo-planar imaging sequence (echo time TE = 77 ms, repetition time TR = 3300 ms) with 64 diffusion directions acquired twice (measurements for each direction = 2), with the diffusion weighting of b = 1000 s/mm^2^ and 11 B0 volume, 1.35 × 1.35 × 2 mm voxels, acquisition matrix = 130 × 130, and 48 slices per volume.

#### 2.3.2. Data Processing

After correcting for distortions caused by susceptibility, eddy currents, and head motion, MD and FA maps were computed. DTI data were adjusted by applying an affine transformation of each image to the B0 image using the Oxford FSL toolkit (available online: https://fsl.fmrib.ox.ac.uk accessed on 12 March 2019). For each voxel, tensor eigen vectors and corresponding eigen values, as well as FA and MD values, were calculated. FA and MD maps were then applied into a standard TBSS (tract-based spatial statistic) skeleton using the Oxford FSL toolkit [24]. Next, each subject’s FA and MD data were mapped onto FMRIB58_FA standard space (which is in the same space as the MNI152 standard space). The quality of this registration was visually checked for each subject’s FA and MD maps. Then, TBSS skeletons were divided according to the John Hopkins ICBM-DTI-81 atlas containing 48 white matter tracts. For each region, average FA and MD, and corresponding AD (axial diffusivity) and RD (radial diffusivity) values, were extracted.

#### 2.3.3. Statistical Analysis 

Averaged FA and MD values in the ROI were compared between NS and HC samples using a standard non-parametric test. False discovery rate (FDR) *p* values were computed using Freeman & Lane (1983) (default in FSL GLM randomize) to control the FDR for multiple tests.

### 2.4. Region-Based Morphometry

Fifty eight (34—NS, 24—HC) individuals took part in this part of the examination. 

Brain-region group differences were analyzed using the CAT12 toolbox (C. Gaser, Structural Brain Mapping Group, Jena University Hospital, Jena, Germany; http://dbm.neuro.uni-jena.de/cat/ (accessed on 20 October 2015)) under SPM12. We used standard-protocol (http://dbm.neuro.uni-jena.de/cat12/CAT12-Manual-old.pdf, accessed on 20 October 2015) with default parameters for segmentation, surface estimation, data resampling, and smoothing. Subsequently, we extracted cortical surface thickness parameters from 150 cortical atlas locations developed by Destrieux (h.aparc.a2009s.annot) [25]. Then, multiple two-sample Wilcoxon rank sum exact tests were carried out to compare the cortical thicknesses of analyzed brain regions between groups. We applied FDR in order to correct for multiple comparisons. 

## 3. Results

### 3.1. Cognitive Evaluation

Intellectual functioning varied widely among participants with NS, ranging from moderately impaired (IQ = 45) to high average (IQ = 121). 

**Figure 1:**
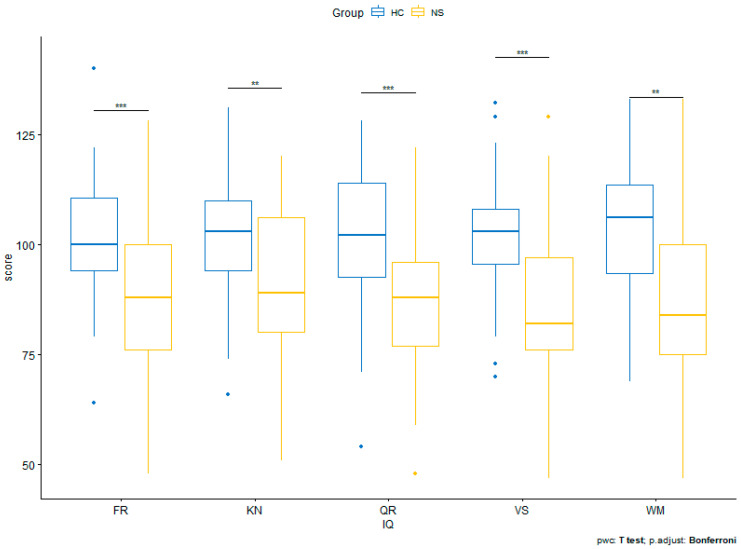
Performance of patients with Noonan syndrome and HC on cognitive measures. Note. ** *p* < 0.01. *** *p* < 0.001.

The analysis revealed a statistically significant effect for the group: F(1.62) = 16.164; *p* < 0.001. Post hoc tests with Bonferroni correction showed that subjects with Noonan syndrome scored significantly lower than healthy subjects on all intelligence dimensions. There was no main effect for the intelligence domain (F(4.248) = 0.198; *p* = 0.939), nor was there an interaction effect between within-group and between-subject factors; F(4.248) = 0.615; *p* = 0.652. The results are shown in Table 1 and Figure 1.

**Figure 2:**
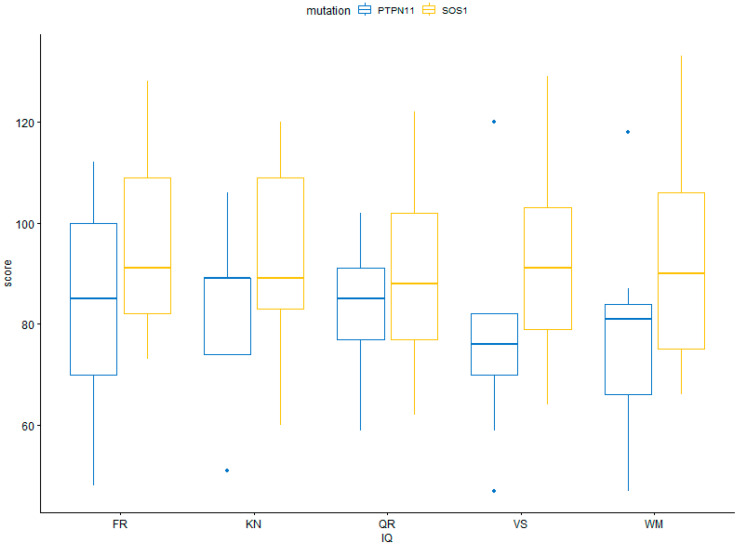
Performance of patients with *PTPN11* and *SOS1* mutations on cognitive measures.

Further, patients with the *PTPN11* mutation scored lower in all cognitive measures compared to patients with the *SOS1* mutation, but differences were not statistically significant, probably due to the small group size. The results are shown in Table 2 and Figure 2.

### 3.2. MRI Examination of the Brain Structures

Anatomical analysis conducted by the radiologist did not reveal any structural anomaly in the brain or posterior cranial cavity for all but one patient with NS.

### 3.3. DTI Results

**Figure 3:**
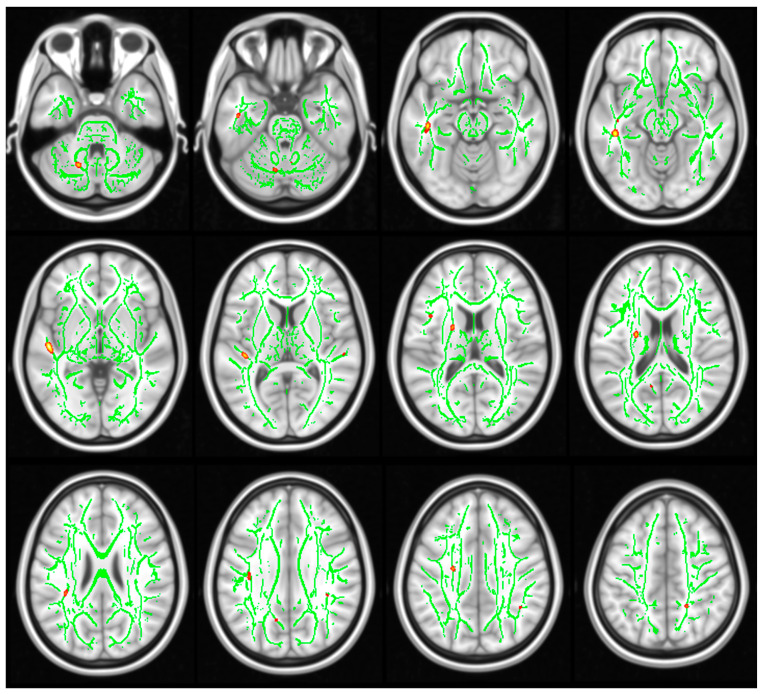
Differences in mean diffusivity (MD) between the subgroups of patients with Noonan syndrome (NS) and healthy controls (HC). Red: increased MD and green: FA skeleton. Results are corrected for multiple comparisons at FWE, *p*  <  0.05. Images are shown according to radiological orientation.

**Figure 4:**
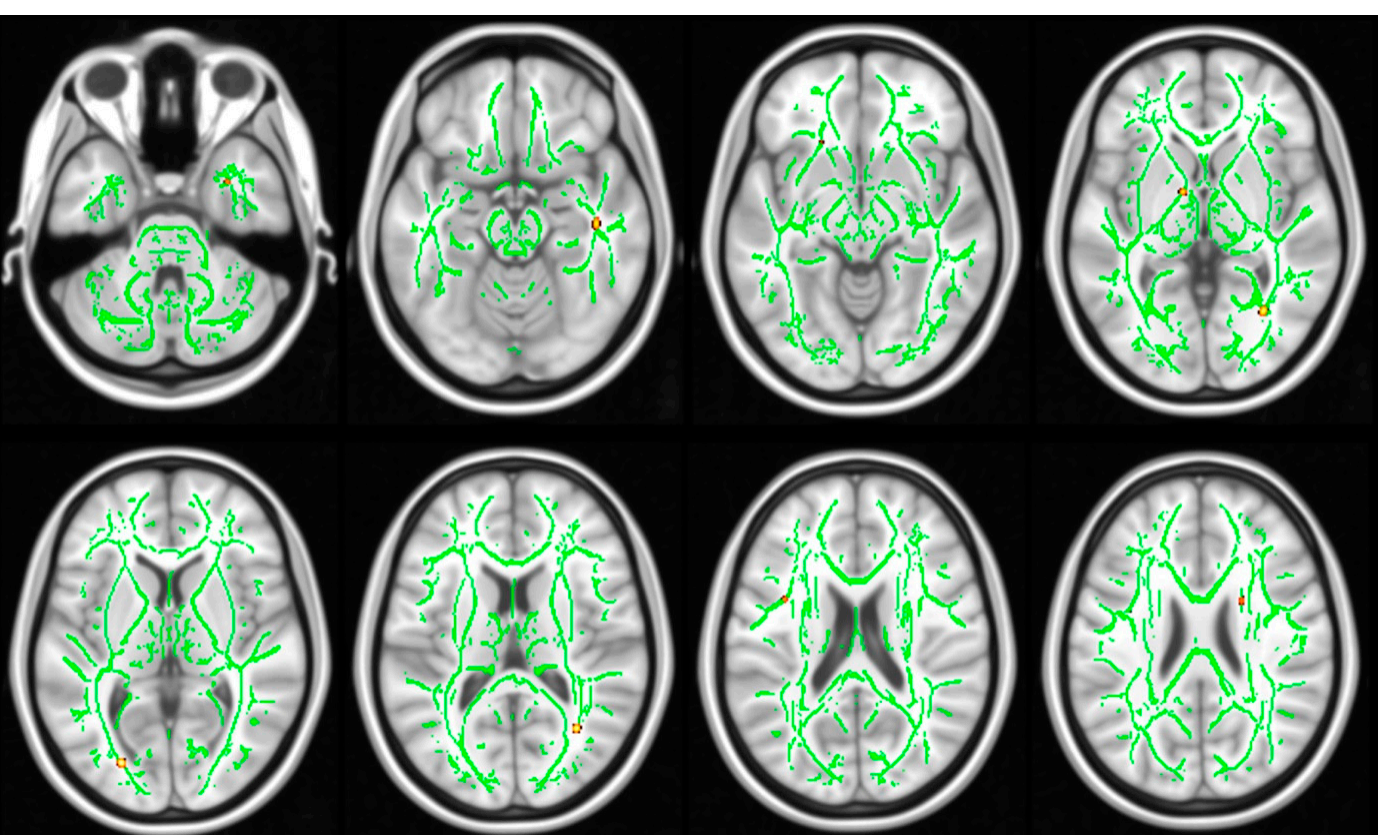
Differences in fractional anisotropy (FA) between the subgroups of healthy controls (HC) and NS. Red: increased FA in HC in comparison to NS. Results are corrected for multiple comparisons at FWE, *p*  <  0.05. Images are shown according to radiological orientation.

DTI metrics yielded significant differences between groups, demonstrating higher MD (Figure 3) and lower FA (Figure 4) in subjects with NS in comparison with the healthy controls. 

In particular, there was lower FA in the following tracts: anterior limb of internal capsule R (*p* = 0.014), anterior limb of internal capsule L (*p* = 0.008), posterior limb of internal capsule R (*p* = 0.034), posterior limb of internal capsule L (*p* = 0.012), retrolenticular part of internal capsule L (*p* = 0.046), anterior corona radiata L (*p* = 0.002), superior corona radiata R (*p* = 0.036), posterior thalamic radiation (including optic radiation) R (*p* = 0.002), sagittal stratum (include inferior longitudinal fasciculus and inferior fronto-occipital fasciculus) R (*p* = 0.040), external capsule R (*p* = 0.020), external capsule L (*p* = 0.012), superior longitudinal fasciculus L (*p* = 0.034), and superior fronto-occipital fasciculus (could be a part of anterior internal capsule) R (*p* = 0.020); and higher MD in: middle cerebellar peduncle (*p* = 0.002), genu of corpus callosum (*p* = 0.014), body of corpus callosum (*p* = 0.010), splenium of corpus callosum (*p* = 0.024), medial lemniscus L (*p* = 0.044), inferior cerebellar peduncle R (*p* = 0.042), inferior cerebellar peduncle L (*p* = 0.002), anterior limb of internal capsule L (*p* = 0.002), posterior limb of internal capsule L (*p* = 0.002), retrolenticular part of internal capsule L (*p* = 0.004), anterior corona radiata R (*p* = 0.004), anterior corona radiata L (*p* = 0.002), superior corona radiata R (*p* = 0.024), superior corona radiata L (*p* = 0.002), posterior corona radiata R (*p* = 0.044), posterior corona radiata L (*p* = 0.020), posterior thalamic radiation (include optic radiation) R (*p* = 0.024), posterior thalamic radiation (including optic radiation) L (*p* = 0.002), sagittal stratum (including inferior longitidinal fasciculus and inferior fronto-occipital fasciculus) R (*p* = 0.024), sagittal stratum (including inferior longitidinal fasciculus and inferior fronto-occipital fasciculus) L (*p* = 0.002), external capsule R (*p* = 0.022), external capsule L (*p* = 0.004), cingulum (cingulate gyrus) L (*p* = 0.048), fornix (cres)/stria terminalis (cannot be resolved with current resolution) L (*p* = 0.018), superior longitudinal fasciculus R (*p* = 0.002), superior longitudinal fasciculus L (*p* = 0.002). 

### 3.4. Rs-FMRI Results

**Figure 5:**
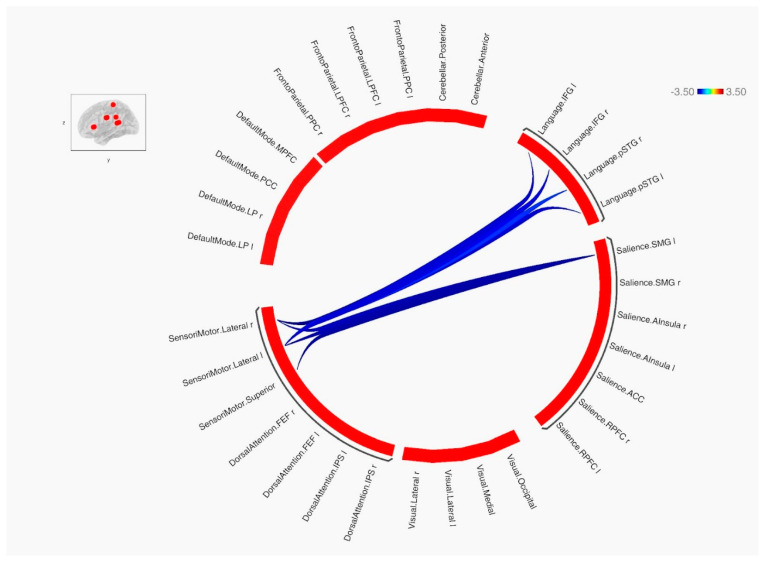
Mean FC in HC > NS presented as a connectome ring and a connectivity matrix showing nodes with decreased FC (blue).

**Figure 6:**
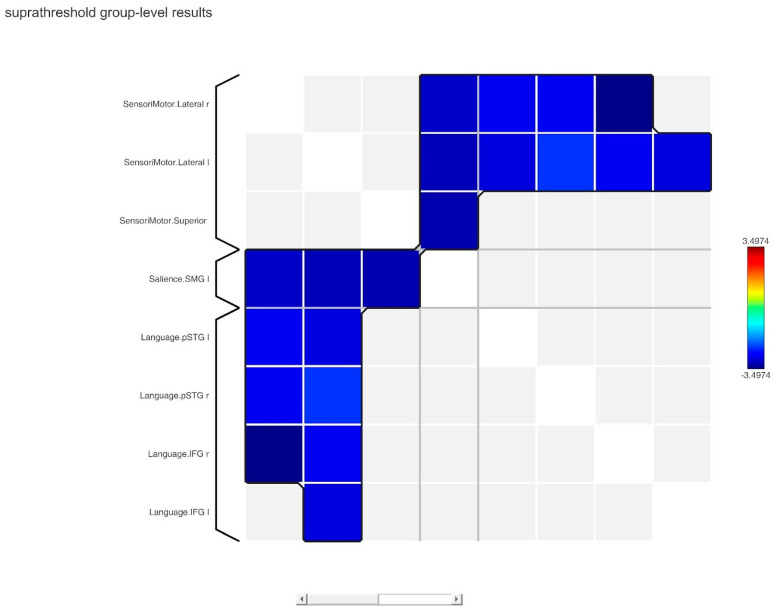
Connectome matrix network correlations computed using functional network connectivity (FNC).

The ROI–ROI FC analysis using the SPC method revealed a significant decrease in inter-network brain FC within one cluster in the HC group in comparison to the NS group (Table 3, Figure 5 and Figure 6). 

### 3.5. Region-Based Morphometry Results

Compared to the HC group, patients with Noonan syndrome showed decreased cortical thickness in the left gyrus rectus (*p* < 0.05 FDR). 

## 4. Discussion

The neurobiological mechanisms leading to cognitive deficits in some patients with Noonan syndrome are not yet well known. However, experimental animal models have suggested a possible association with molecular defects within the RAS/MAPK signal transduction pathway [12]. 

Fattah et al. examined children with Noonan syndrome (*n* = 17, mean age range 8.68 ± 2.39) using different DTI scalar measures as well as structural MRI. The analysis, published in 2021, revealed widespread reductions in white matter connectivity in these patients [26]. Johnson et al. evaluated children with molecularly confirmed Noonan syndrome (*n* = 12, age range 4–11 years) using high-resolution MRI scans and found subcortical and cortical differences between patients with Noonan syndrome and healthy controls [27].

The aforementioned studies reveal that weaknesses in cognitive functioning and neuroimaging alterations are much more frequent in patients with Noonan syndrome than in the general population. It is highly plausible that cognitive deficits, as well as differences in brain structures, might be associated with mutations in particular genes leading to a dysregulation of the RAS/MAPK pathway.

In this study, cognitive abilities of patients with Noonan syndrome were assessed using the Stanford Binet Intelligence Scales (Fifth Edition, SB5). NS patients demonstrated decreased verbal and nonverbal IQ, fluid reasoning, knowledge, quantitative reasoning, visual–spatial processing, and working memory compared to healthy controls. In addition, we observed discrepancies in the cognitive functioning of individuals with mutations in *PTPN11* and *SOS1.* Specifically, individuals with mutations in *PTPN11* showed lowered cognitive functioning compared to those with mutations in *SOS1*. These results are in line with previous studies [11,28].

Neurodevelopmental anomalies in specific brain regions have been suggested to be related with cognitive impairments in patients with Noonan syndrome. In order to obtain reliable information on brain function region-based morphometry (RBM), white matter connectivity measurements and Rs-fMRI data acquisition were performed.

Only one patient in our study (a 5-year-old boy) was diagnosed with structural brain abnormality (Arnold-Chiari I malformation) without any neurological symptoms. Anatomical brain defects are not a common finding in Noonan syndrome. Individuals with Noonan syndrome and with Arnold-Chiari malformation have rarely been reported [29,30].

In contrast to anatomical analysis, DTI metrics revealed significant differences between groups, demonstrating higher MD and lower FA in NS patients in comparison to healthy controls. Anatomical differences between subjects with NS and HC (revealed by alternations of gray matter thickness and diffusion parameters characterizing the brain’s white matter) were widely distributed throughout the brain, indicating holistic, non-specific changes, possibly resulting from Noonan Syndrome. These outcomes correspond with the cognitive assessment demonstrating impaired functioning in all domains without indicating predominant difficulty in any particular area of cognitive functioning.

ROI–ROI rsFC revealed hyper-connectivity in NS patients between the sensorimotor network and language network and between the sensorimotor network and salience network. These result are congruent with the neuropsychological profile of NS patients observed in our study. Lower verbal IQ might be attributable (at least partially) to altered functional connectivity between regions comprising the language network and sensorimotor network, with such connectivity involved in executing appropriate motor responses. Considering the fact that the salience network is critical in detecting and filtering salient stimuli [31], its atypical connectivity with the sensorimotor network might be related to the decreased psychomotor response to relevant environmental stimuli observed in subjects with NS, as shown in previous studies [32].

Accurate evaluation of neuropsychological profiles and underlying possible functional and structural abnormalities (including the assessment of microstructural white matter alterations as well as rsFC analysis) in Noonan syndrome patients gives us a better understanding of the essential impact of the RAS/MAPK signaling pathway’s dysregulation on human brain function. However, further analysis is needed in a larger cohort for a more precise characterization of the mechanisms responsible for cognitive and neurofunctioning impairments.

While the present findings provide insights into the behavioral phenotype of RASopathies, further studies including more specific behavioral assessments in larger and more homogeneous patient cohorts are required for a more precise characterization of phenotypes associated with each disorder, individual disease genes, and types of molecular lesions.

## Figures and Tables

**Table 1:** Performance of patients with Noonan syndrome and HC on cognitive measures.

Variables	Group	Min	Max	M	SD	Me
IQ	NS	45.00	121.00	84.81	17.87	86.00
	HC	64.00	135.00	102.56	16.84	105.00
Verbal IQ	NS	46.00	121.00	85.54	17.11	84.00
	HC	73.00	135.00	101.15	14.70	100.00
Nonverbal IQ	NS	47.00	122.00	86.27	17.93	87.00
	HC	50.00	139.00	103.52	20.78	109.00
Fluid Reasoning	NS	48.00	128.00	86.92	18.20	88.00
	HC	64.00	140.00	102.07	15.09	100.00
Knowledge	NS	51.00	120.00	89.68	17.35	89.00
	HC	66.00	131.00	101.07	14.84	103.00
Quantitative Reasoning	NS	48.00	122.00	86.24	15.43	88.00
	HC	54.00	128.00	101.44	17.97	102.00
Visual Spatial Processing	NS	47.00	129.00	86.32	17.95	82.00
	HC	70.00	132.00	103.11	17.01	103.00
Working Memory	NS	47.00	133.00	87.19	19.86	84.00
	HC	69.00	133.00	102.85	16.72	106.00

NS—Patients with Noonan Syndrome; HC—Healthy Controls.

**Table 2:** Performance of patients with *PTPN11* and *SOS1* mutations on cognitive measures.

Variables	Group	Min	Max	M	SD	Me
IQ	*PTPN11*	45	113	75.778	21.028	79
	*SOS1*	64	121	91.556	20.427	91
Verbal IQ	*PTPN11*	47	120	79.444	22.445	79
	*SOS1*	66	122	93.889	18.651	91
Nonverbal IQ	*PTPN11*	46	105	75.556	17.896	78
	*SOS1*	62	121	90.556	20.58	86
Fluid Reasoning	*PTPN11*	48	112	82.444	22.683	85
	*SOS1*	73	128	95.444	17.854	91
Knowledge	*PTPN11*	51	106	80.333	18.815	89
	*SOS1*	60	120	94.444	20.335	89
Quantitative Reasoning	*PTPN11*	59	102	82.889	12.201	85
	*SOS1*	62	122	89.556	18.447	88
Visual Spatial Processing	*PTPN11*	47	120	76.778	20.042	76
	*SOS1*	64	129	91.778	19.556	91
Working Memory	*PTPN11*	47	118	76.222	21.896	81
	*SOS1*	66	133	93.667	23.211	90

*PTPN11*—Patients with *PTPN11* mutation; *SOS1*—Patients with *SOS1* mutation.

**Table 3:** Statistical cluster analysis of the networks using spatial pairwise clustering (SPC) presenting decreased connectivity between nodes.

			p-unc	p-FDR	p-FWE
Cluster 1		Score = 39.00	0.010959	0.120546	0.051000
		Mass = 164.31	0.001234	0.013575	0.007000
		Size = 18	0.000746	0.008202	0.003000
SensoriMotor.Lateral r	Language.IFG r	T(48) = −3.50	-	0.001023	0.140478
SensoriMotor.Superior	Salience.SMG l	T(48) = −3.24	-	0.002180	0.140478
SensoriMotor.Lateral l	Salience.SMG l	T(48) = −3.16	-	0.002724	0.140478
SensoriMotor.Lateral r	Salience.SMG l	T(48) = −3.07	-	0.003495	0.140478
SensoriMotor.Lateral l	Language.pSTG l	T(48) = −2.92	-	0.005250	0.140478
SensoriMotor.Lateral l	Language.IFG l	T(48) = −2.92	-	0.005381	0.140478
SensoriMotor.Lateral r	Language.pSTG l	T(48) = −2.77	-	0.007879	0.145402
SensoriMotor.Lateral r	Language.pSTG r	T(48) = −2.77	-	0.008030	0.145402
SensoriMotor.Lateral l	Language.IFG r	T(48) = −2.76	-	0.008208	0.145402

Note: p-unc—uncorrected *p*-value, p-FDR—false discovery rate adjusted *p*-value, p-FWE—family-wise error rate adjusted *p*-value, r—right, l—left.

## Data Availability

The data presented in this study are available on request from the corresponding author. The data are not publicly available due to their size.

## References

[1] Johnston J.J., van der Smagt J.J., Rosenfeld J.A., Pagnamenta A.T., Alswaid A., Baker E.H., Blair E., Borck G., Brinkmann J., Craigen W. (2018). Autosomal recessive Noonan syndrome associated with biallelic LZTR1 variants. Genet. Med..

[2] Bezniakow N., Gos M., Obersztyn E. (2014). The RASopathies as an example of RAS/MAPK pathway disturbances—Clinical presentation and molecular pathogenesis of selected syndromes. Dev. Period. Med..

[3] Carcavilla A., Suárez-Ortega L., Rodríguez Sánchez A., Gonzalez-Casado I., Ramón-Krauel M., Labarta J.I., Quinteiro Gonzalez S., Riaño Galán I., Ezquieta Zubicaray B., López-Siguero J.P. (2020). Noonan syndrome: Genetic and clinical update and treatment options. An. Pediatr. Engl. Ed..

[4] Lee B.H., Yoo H.-W. (2019). Noonan syndrome and RASopathies: Clinical features, diagnosis and management. J. Genet. Med..

[5] Tidyman W.E., Rauen K.A. (2009). The RASopathies: Developmental syndromes of Ras/MAPK pathway dysregulation. Curr. Opin. Genet. Dev..

[6] Gómez N., Cohen P. (1991). Dissection of the protein kinase cascade by which nerve growth factor activates MAP kinases. Nature.

[7] Raman M., Chen W., Cobb M.H. (2007). Differential regulation and properties of MAPKs. Oncogene.

[8] van der Burgt I., Thoonen G., Roosenboom N., Assman-Hulsmans C., Gabreels F., Otten B., Brunner H.G. (1999). Patterns of cognitive functioning in school-aged children with Noonan syndrome associated with variability in phenotypic expression. J. Pediatr..

[9] Alfieri P., Piccini G., Caciolo C., Perrino F., Gambardella M.L., Mallardi M., Cesarini L., Leoni C., Leone D., Fossati C. (2014). Behavioral profile in RASopathies. Am. J. Med. Genet. A.

[10] Pierpont E.I., Tworog-Dube E., Roberts A.E. (2015). Attention skills and executive functioning in children with Noonan syndrome and their unaffected siblings. Dev. Med. Child. Neurol..

[11] Pierpont E.I., Pierpont M.E., Mendelsohn N.J., Roberts A.E., Tworog-Dube E., Seidenberg M.S. (2009). Genotype differences in cognitive functioning in Noonan syndrome. Genes. Brain Behav..

[12] Cesarini L., Alfieri P., Pantaleoni F., Vasta I., Cerutti M., Petrangeli V., Mariotti P., Leoni C., Ricci D., Vicari S. (2009). Cognitive profile of disorders associated with dysregulation of the RAS/MAPK signaling cascade. Am. J. Med. Genet. A.

[13] Nemoto K. (2017). Understanding Voxel-Based Morphometry. Brain Nerve..

[14] Kakeda S., Korogi Y. (2010). The efficacy of a voxel-based morphometry on the analysis of imaging in schizophrenia, temporal lobe epilepsy, and Alzheimer’s disease/mild cognitive impairment: A review. Neuroradiology.

[15] Chao T.C., Chou M.C., Yang P., Chung H.W., Wu M.T. (2009). Effects of interpolation methods in spatial normalization of diffusion tensor imaging data on group comparison of fractional anisotropy. Magn. Reson. Imaging.

[16] Meyer-Lindenberg A. (2012). The future of fMRI and genetics research. Neuroimage.

[17] Pluta A., Wolak T., Czajka N., Lewandowska M., Cieśla K., Rusiniak M., Skarżyński H. (2014). Reduced resting-state brain activity in the default mode network in children with (central) auditory processing disorders. Behav. Brain Funct. BBF.

[18] Wang Q., Li H.Y., Li Y.D., Lv Y.T., Ma H.B., Xiang A.F., Jia X.Z., Liu D.Q. (2021). Resting-state abnormalities in functional connectivity of the default mode network in autism spectrum disorder: A meta-analysis. Brain Imaging Behav..

[19] van der Burgt I. (2007). Noonan syndrome. Orphanet J. Rare Dis..

[20] Roid G.H., Sajewicz-Radtke U., Radtke B.M., Lipowska M. (2017). Stanford-Binet Intelligence Scales.

[21] R Core Team (2021). R: A Language and Environment for Statistical Computing.

[22] Ho D., Imai K., King G., Stuart E. (2011). MatchIt: Nonparametric Preprocessing for Parametric Causal Inference. J. Stat. Softw..

[23] Zalesky A., Cocchi L., Fornito A., Murray M.M., Bullmore E. (2012). Connectivity differences in brain networks. Neuroimage.

[24] Smith S.M., Jenkinson M., Woolrich M.W., Beckmann C.F., Behrens T.E., Johansen-Berg H., Bannister P.R., De Luca M., Drobnjak I., Flitney D.E. (2004). Advances in functional and structural MR image analysis and implementation as FSL. Neuroimage.

[25] Destrieux C., Fischl B., Dale A., Halgren E. (2010). Automatic parcellation of human cortical gyri and sulci using standard anatomical nomenclature. Neuroimage.

[26] Fattah M., Raman M.M., Reiss A.L., Green T. (2021). *PTPN11* Mutations in the Ras-MAPK Signaling Pathway Affect Human White Matter Microstructure. Cereb. Cortex..

[27] Johnson E.M., Ishak A.D., Naylor P.E., Stevenson D.A., Reiss A.L., Green T. (2019). *PTPN11* Gain-of-Function Mutations Affect the Developing Human Brain, Memory, and Attention. Cereb. Cortex..

[28] Wingbermühle E., Roelofs R.L., Oomens W., Kramer J., Draaisma J.M.T., Leenders E., Kleefstra T., Kessels R.P.C., Egger J.I.M. (2022). Cognitive Phenotype and Psychopathology in Noonan Syndrome Spectrum Disorders through Various Ras/MAPK Pathway Associated Gene Variants. J. Clin. Med..

[29] Keh Y.S., Abernethy L., Pettorini B. (2013). Association between Noonan syndrome and Chiari I malformation: A case-based update. Childs Nerv. Syst..

[30] Ejarque I., Millán-Salvador J.M., Oltra S., Pesudo-Martínez J.V., Beneyto M., Pérez-Aytés A. (2015). Arnold-Chiari malformation in Noonan syndrome and other syndromes of the RAS/MAPK pathway. Rev. Neurol..

[31] Menon V., Toga A.W. (2015). Salience network. Brain Mapping: An Encyclopedic Reference.

[32] Wingbermühle E., Roelofs R.L., van der Burgt I., Souren P.M., Verhoeven W.M., Kessels R.P., Egger J.I. (2012). Cognitive functioning of adults with Noonan syndrome: A case-control study. Genes. Brain Behav..

